# A Novel Metal-Organic Framework Composite, MIL-101(Cr)@MIP, as an Efficient Sorbent in Solid-Phase Extraction Coupling with HPLC for Tribenuron-Methyl Determination

**DOI:** 10.1155/2019/2547280

**Published:** 2019-06-02

**Authors:** Xinyuan Zheng, Junping Wang

**Affiliations:** ^1^State Key Laboratory of Food Nutrition and Safety, Tianjin University of Science & Technology, Tianjin 300457, China; ^2^Tianjin Institute for Drug Control, Tianjin 300070, China

## Abstract

A highly efficient and selective method based on core–shell molecularly imprinted polymers (MIL@MIP) and high performance liquid chromatography (HPLC) was developed and firstly used for the trace analysis of tribenuron-methyl (TBM) in complicated matrices. The MIL@MIP was prepared by surface molecular-imprinting technique, specially using MIL-101 as core, TBM as template molecule, methacrylic acid (MAA) as functional monomer, ethylene glycol dimethacrylate (EGDMA) as cross-linker, and azobisisobutyronitrile (AIBN) as initiator. The resulting MIL@MIP showed high affinity, recognition specificity, fast mass transfer rate, and efficient adsorption performance towards TBM with the adsorption capacity reaching up to 3.217 mg/g. It also showed high cross-selectivity for TBM among its six kinds of chemical structure analogues. Furthermore, using the MIL@MIP as solid-phase extraction (SPE) materials, the recoveries of TBM determined by HPLC were 84.6-92.3%, 93.3-106.7%, and 88.9-93.3% in the spiked river water, soil, and soybean samples, respectively, with the limit of detection of 0.3 ng/L, 1.5 ng/kg, and 1.5 ng/kg, accordingly. It was proved that the developed HPLC-MISPE method was fast, accurate, and sensitive for detecting the trace TBM in river water, soil, and soybean samples.

## 1. Introduction

Tribenuron-methyl (TBM) is one of the sulfonylurea herbicides (SUs), which have been widely used to control broad-leaved weeds and annual grasses due to their high herbicidal activity, low dosage (4-20g of active ingredient per hectare), and relatively low mammalian toxicity (LD_50_> 4000 mg/kg) [1, 2]. As a widely used herbicide, its increasing application and abuse may result in the accumulation in real samples such as soil, river, and crop samples. More and more attention has been paid to its toxicity on crops and human health [3]. China has also set the maximum residue limits (MRLs) for TBM in cereals as 0.05 mg/kg [4]. It is a big challenge for us to enrich and separate TBM, for its extremely low residual as well as being mixed with complex matrix. Up to now, different methods have been developed to determine TBM residues, including high performance liquid chromatography with ultraviolet detector (HPLC-UV) [5], high performance liquid chromatography with mass spectrum (HPLC/MS/MS) [6, 7], capillary electrophoresis (CE) [8–10], and enzyme-linked immunoassay (ELISA) [11]. Along with these methods, most sample pretreatments for sulfonylurea analysis used solid-phase extraction (SPE) with C_18_ columns for purification and concentration. However, these pretreatment methods are time-consuming and lack selectivity of the target objects, leading to serious interference with matrix during the actual application. Therefore, a highly selective material and efficient extraction before quantitative determination are essentially necessary.

The molecular-imprinting technique is a recently developed method for producing polymeric macromolecules with specific recognition sites complementary in shape, size, and functional groups to template molecules. With the advantages of high selectivity, structure stability, easy preparation, and low cost, the MIP have been used in many aspects, such as vitro diagnostics [12, 13], drug delivery [14, 15], food safety [16, 17], and receptors in sensors [18–20]. In recent years, there have been reports on the use of MIP for TBM detection [21–23]. However, these methods exhibited relatively slow mass transfer, small binding capacity, and low recovery to some particular components. To address these issues, the surface molecular-imprinting strategy by immobilization of template molecules at the surface of solid substrates has been recently introduced, which was conducted by immobilization of MIP at the surface of the core, such as silica nanoparticles [24], multiwalled carbon nanotubes [25], and magnetic nanoparticles [26]. Compared with these materials used as the core of MIP, metal-organic framework (MOF) showed great advantages, such as large surface area, good hydrothermal stability, uniform cavities, and superior adsorption capacity [27–29]. Up to now, there is no report particularly concerned with TBM detection using the hybrid material of MOFs and MIP as sorbents. In the current studies about the residue detection of SUHs, the recovery was usually bad for TBM [30]. In fact, TBM has great phytotoxicity even at low concentrations [31].

In this paper, we prepared a kind of MIL@MIP polymer on the surface of MIL-101 using TBM as the template molecule. The morphology and adsorption ability of the obtained polymer were characterized, which exhibited high adsorbing capacity and high cross-selectivity. Furthermore, the obtained polymer was firstly used as SPE materials to detect TBM residue and it exhibited good recoveries in spiked river water, soil, and soybeans, which showed great potential for detecting the trace TBM in samples with complex matrix.

## 2. Materials and Methods

### 2.1. Reagent and Instrument

**Figure 1:**
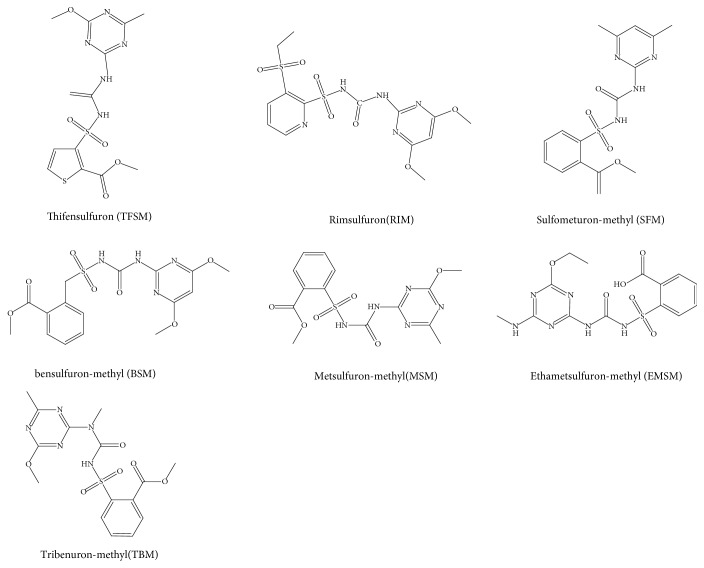
Chemical structure of tribenuron-methyl and analogues.

Thifensulfuron methyl (TFSM,97.5%, M_w_=387.39), rimsulfuron (RIM, 99.9%, M_w_=431.44), sulfometuron methyl (SFM, 99.9%, M_w_= 364.38), bensulfuron-methyl (BSM, 99.6%, M_w_=410.40), metsulfuron-methyl (MSM, 99.2%, M_w_=381.36), ethametsulfuron-methyl (EMSM, 98.7%, M_w_=410.41), and tribenuron-methyl (TBM, 99.4%, M_w_=395.39) were purchased from the Sigma Group (USA), and their structures are shown in Figure 1. Methacrylic acid (MAA), dimethyl formamide (DMF), and toluene were provided by the Guoyao Group Reagent, Co. (Shanghai, China). Ethylene glycol dimethacrylate (EGDMA) was provided by Aldrich (Deisenhofen, Germany); 2,2-azobisisobutyronitrile (AIBN, >98%) was obtained from the No. 4 Reagent & H. V. Chemical Co. Ltd. (Shanghai, China); HPLC grade acetonitrile and methanol were purchased from Merck & Co. All other chemicals were of analytical grade.

The resulting MIL-101 and MIP@MIL were characterized by Hitachi S-3000N scanning electron microscope (SEM, Tokyo, Japan), Vector 22 FT-IR spectrophotometer (Bruker, USA), and D2 PHASER X-ray diffraction (XRD) (Bruker, USA). Chromatographic system consists of Agilent 1200 quaternary pumps and DAD detector (Agilent, USA). HPLC ChemStation software (Agilent, USA) was used for the data analysis. HPLC analysis was carried out with an HPLC system (Agilent 1200, USA) and Agilent SB-C_18_ column (4.6 mm × 250 mm, 5 *μ*m) at a mobile phase consisted of acetonitrile and phosphoric solution (pH=2.5) (40 : 60), and the flow rate was 1.0mL/min. The detection wavelength was 230 nm and the acquired spectral and chromatographic data was processed by Agilent ChemStation software.

### 2.2. Preparation of MIL-101 and MIL@MIP

#### 2.2.1. Preparation of MIL-101

MIL-101 was prepared as the report [32]: Cr(NO_3_)_3_·9H_2_O (4.01 g, 10 mmol), 1,4-benzenedicarboxylic acid (H_2_BDC, 1.64 g, 10 mmol) and hydrofluoric acid (HF, 125 *μ*L, concentration: 40%) were dissolved in 70 ml of ultrapure water by ultrasonication. Then the mixture was transferred to high pressure reactor at 220°C for 8h, followed by cooling at room temperature. After being centrifuged (4000 r/min, 15 minutes), the precipitate was washed three times with ultrapure water by sonication for 10 minutes. The green powder product MIL-101 was then obtained after being dried under vacuum.

#### 2.2.2. Preparation of MIL@MIP

**Figure 2:**
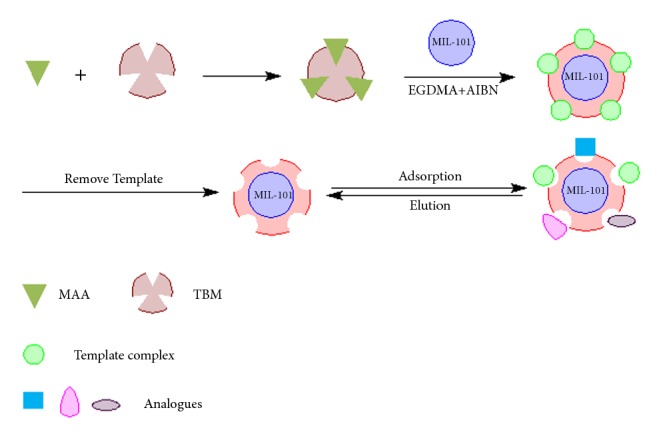
Process for preparing of MIL@MIP.

As illustrated in Figure 2, firstly, the template complex was formed between MAA and template molecules based on the interaction of *π*-*π* stacking and intermolecular hydrogen. Then the template complex was copolymerized uniformly at the surface of MIL-101 core with the help of EGDMA and AIBN. At last, the MIL@MIP were achieved after the template molecules were removed. Its detailed procedures were as follows: 395.40 mg (1.0 mmol) of molecule template (TBM) was dissolved in 4.0 mL dimethyl formamide and 2.0 mL toluene by magnetic stirring; 0.28 mL of MAA (3.0 mmol) was added when the molecule template was dissolved and then mixed with 1.32 mL of EGDMA (7.0 mmol) after 20 mg of MIL-101 was added. The mixture was stirred for 30 min before 60 mg of cross-linker AIBN was added. After being stirred for another 5 min and protected by nitrogen for 10 min, the mixture was sealed and thermally initiated at 60°C for 24 h. After the polymerization, the template was removed by Soxhlet extraction with 250 mL of methanol/acetic acid (9:1, v/v) and 250 ml of methanol in sequence until no analyte was detected by HPLC.

For comparison, a metal-organic framework nonimprinted polymer (MIL@NIP) was also prepared by the same way in the absence of the template.

### 2.3. Adsorption Isotherm Experiment

Static equilibrium adsorption experiments were conducted and repeated five times: 20 mg of MIL@MIP or MIL@NIP was put into the 50 mL glass volumetric flask, containing 20 mL of methanol solution with TBM concentration varying from 6.26 to 100 *μ*g/mL, and shaken at the speed of 200 rpm for 90 minutes at room temperature. The supernatant and polymer were separated by the nylon filter, and the TBM concentration in the supernatant was measured by HPLC. According to the TBM concentration before and after adsorption, the equilibrium amount of substrate bound to the polymer (Q, mg/g) was calculated by the following equation [33]:

(1) $$\begin{array}{c} Q=\frac{V\left(C_{0}-C_{1}\right)}{m} \end{array}$$

where C_0_ and C_1_ represent the initial concentration and final concentration (*μ*g/mL) of TBM, respectively; V (mL) represents the volume of solution; m (mg) is the weight of the polymer; and Q is the adsorption amount of TBM. In order to evaluate and analyze the binding properties of MIL@MIP, experimental data were fitted to Langmuir model (2) [34]:

(2) $$\begin{array}{c} \frac{\left[TBM\right]}{Q}=\frac{1}{\left(Q_{max}K_{D}\right)}+\frac{\left[TBM\right]}{Q_{max}} \end{array}$$

where Q represents the adsorption amount of the polymer (mg/g), [TBM] represents residual concentration (*μ*g/mL) of TBM at equilibrium adsorption, Q_max_ is the maximum adsorption amount of the polymer (mg/g), K_D_ is the dissociation constant, and values of K_D_ and Q_max_ can be calculated from the slope and intercept of lines fitted by [TBM]/Q to [TBM].

### 2.4. Adsorption Kinetics Experiment

The adsorption kinetics of MIL@MIP was investigated and repeated five times by the following steps: 20 mg of MIL@MIP was put into the 50 mL glass volumetric flask, containing 20 mL of methanol solution with 40 *μ*g/mL TBM, and shaken at the speed of 200 rpm at room temperature. The supernatant was obtained and measured by HPLC at 20 min, 30 min, 50 min, 70min, 90 min, 120 min, and 180 min, respectively, and the amount of substrate bound to the polymer (Q, mg/g) was calculated by (1).

### 2.5. Selectivity Experiment

In order to evaluate the selectivity of MIL@MIP for TBM, the selectivity experiments were conducted and repeated five times: 20 mg of MIL@MIP (or MIL@NIP) was added to the mixed seven-pesticide solution with their initial concentrations of 40 *μ*g/mL, respectively, which were composed of TFSM, RIM, TBM, BSM, MSM, EMSM, and SFM. The mixture with MIL@MIP (or MIL@NIP) was shaken at the speed of 200 rpm at room temperature for 90 minutes. The supernatant was obtained and measured by HPLC, and the interrelated absorbed coefficient was evaluated by the following equation:

the static distribution coefficient:

(3) $$\begin{array}{c} K=\frac{\left(C_{i}-C_{f}\right)\times V}{C_{f}\times M} \end{array}$$

where C_f_ is the concentration of the solution after absorbed, C_i_ is the initial concentration of the solution, V is the volume of the solution, and M is the weight of the MIL@MIP or MIL@NIP.

The selectivity adsorption capacity of MIL@MIP to TBM with respect to the competitor can be evaluated by imprinting factor (IF) and selective factor (SC). The definition of IF and SC is as follows:

(4) $$\begin{array}{c} IF=\frac{K_{i}}{K_{c}} \end{array}$$

where K_i_ represents the static distribution coefficient of MIL@MIP and K_c_ represents the static distribution coefficient of MIL@NIP;

(5) $$\begin{array}{c} SC=\frac{{IF}_{TBM}}{{IF}_{i}} \end{array}$$

where IF_TBM_ is the IF value of MIL@MIP to TBM and IF_i_ is the IF value of MIL@MIP to pesticides TFSM, RIM, BSM, MSM, EMSM, and SFM, respectively.

### 2.6. Separation and Enrichment of TBM

30 mg of MIL@MIP particles was packed into an empty 3mL solid-phase extraction (SPE) cartridge, which was capped with PTFE frits at the top and bottom, respectively. Then it was used as a molecularly imprinted solid-phase extraction (MISPE) and preconditioned with 10 mL methanol and 10 mL water in turn before the use. The standard solution or sample solution was loaded onto the preconditioned MISPE cartridge. After the sample solution passed the MISPE cartridge, the cartridge was washed with 10 mL water and the water eluent was discarded, then the MISPE cartridge was washed with 8 mL acetonitrile, and the obtained acetonitrile extract was collected and blown down under nitrogen to final volume of 1.0 mL for subsequent HPLC analysis. The NISPE was also prepared in the same way using the MIL@ NIP as sorbents.

### 2.7. Sample Pretreatment

The water samples were collected from Haihe River (Tianjin, China) and filtered through 0.22 *μ*m filters. The soil samples (passed through a 2 mm sieve) were collected from the corn fields (Taiyuan, China) and dried under natural conditions. The soybeans were purchased from the local market (Tianjin, China), ground, and passed through a 0.21 mm sieve for the further study.

The river water samples were loaded onto the preconditioned MISPE column after centrifugation. The soil samples and soybean powders were extracted with 10 mL of acetonitrile by homogenizing for 2 min at 12000 rpm and centrifuged for 5 min at 4000 rpm, respectively. The extraction was performed for three times, and the supernatant was concentrated under 40°C by vacuum. The residual was dissolved in 1 mL methanol and then diluted with 20 mL water, and the final solution was utilized to conduct MISPE procedure.

### 2.8. Method Validation

To evaluate accuracy of the method, the recovery tests were performed. The river water samples were spiked with TBM at the final concentrations of 1.3×10^−3^, 6.5×10^−3^, and 1.3×10^−2^*μ*g/L. The soil and soybean samples (1.0g) were prepared using an appropriate volume of TBM standard solution to obtain the final concentrations of 4.5×10^−3^, 2.25×10^−2^, and 4.5×10^−2^*μ*g/kg, respectively. Extraction, purification, and concentration were conducted in the same way mentioned above. Relative standard deviations (RSDs) of measurements were utilized to estimate the precision of the proposed method. The limit of detection (LOD) was also obtained.

## 3. Results and Discussions

### 3.1. Characterization of MIL@MIP

**Figure 3:**
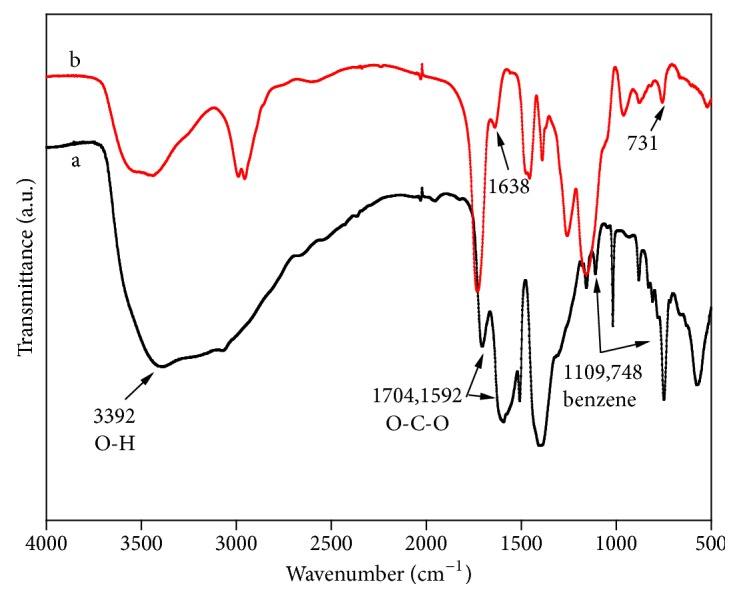
FT-IR spectrum of MIL-101 (a) and MIL@MIP (b).

As was shown in Figure 3(a), strong adsorptions were at the wavelengths of 1592.62cm^−1^ and 1704.72 cm^−1^ caused by the vibration of O-C-O framework, which could verify the existence of dicarboxylic acid organic framework in MIL-101 structure. Besides, a wide adsorption summit on the wavelength of 3391.87cm^−1^ was recorded, which was the very typical summit from the water adsorbing sample surface. The wave summit on 1109.24cm^−1^ and 748.32cm^−1^ was caused by the vibration of benzene ring in bridging ligand. From the above data, it is obvious that MIL-101 was synthesized successfully [35]. As was shown in Figure 3(b), there was O-C-O framework vibration flexibility on 1638 cm^−1^ and 731 cm^−1^, which was similar to Figure 3(a) and proved the existence of MIL-101 structure. Therefore, there was a successful embedding between nucleus MIL-101 and MIP.

**Figure 4:**
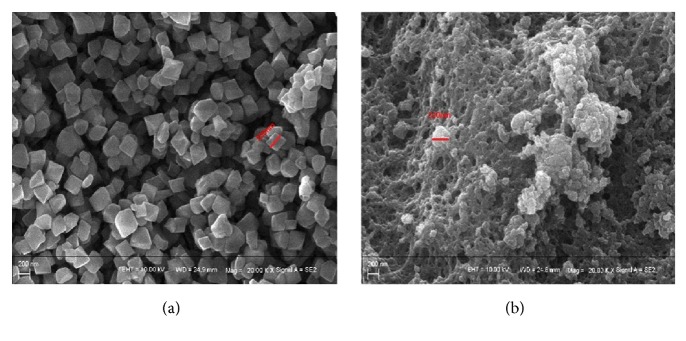
SEM images of MIL-101 (a) and MIL@MIP (b).

SEM was used to observe the morphology of MIL-101 and MIL@MIP. As shown in Figure 4(a), the synthesized MIL-101 crystal particles had almost the same size, relatively smooth surface, and integrated octahedral structure. The size of the crystal particle was about 200 nm. After embedding, the net-and-cavity structure was formed on the surface of the crystal particles, which was shown in Figure 4(b).

**Figure 5:**
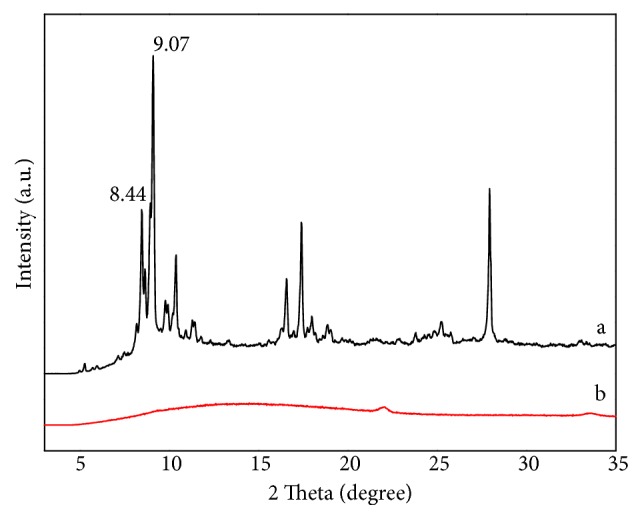
Powder X-ray diffraction for MIL-101 (a) and MIL@MIP (b).

X-Ray diffraction spectra of MIL-101 (*a*) and MIL@MIP (*b*) were shown in Figure 5. The characteristic 2*θ* values of the obtained polymer appeared at 8.44° and 9.07° in Figure 5 (*a*). The location and shape of the summits were in accordance with the report, and its XRD was consistent with the standard spectrum of MIL-101 [36]. As a result, the synthesized crystal particle was MIL-101. As was shown in Figure 5 (*b*), there was no characteristic peak of MIL-101 on XRD spectrum of MIL@MIP, which verified the formation of a nucleus-and-shell structure.

### 3.2. Adsorption Isotherms

**Figure 6:**
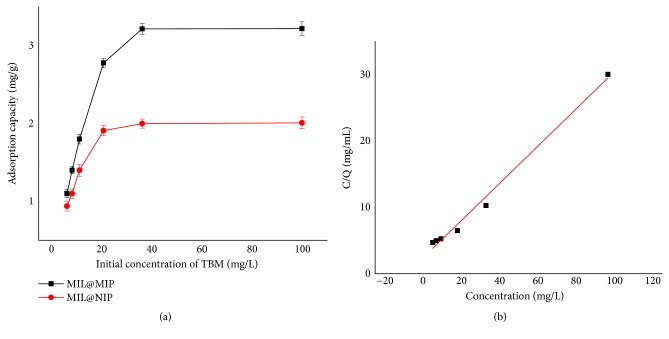
Binding isotherm of MIL@MIP and MIL@NIP for TBM in methanol (a) and Langmuir plot of MIL@MIP (b).

In order to verify the adsorption ability of MIL@MIP synthesized towards TBM, the different concentration of TBM solutions in methanol varying from 6.26 to 100.0 *μ*g/mL were prepared at the room temperature. The adsorption characteristics of MIL@MIP and MIL@NIP were shown in Figure 6(a). The difference in adsorption capacity between MIL@MIP and MIL@NIP was not obvious at the low concentration of TBM. With the increase of TBM concentration, the adsorption capacity of MIL@MIP and MIL@NIP towards TBM also increased to some extent, but the difference between them was more and more clear. When the concentration of TBM was 36.3 *μ*g/mL, the adsorption capacity of MIL@MIP was 3.217 mg/g. At the same time, the adsorption capacity of MIL@NIP was 1.994 mg/g. The adsorption capacity of MIL@MIP was about 1.6 times as strong as that of MIL@NIP. Subsequently, the adsorption capacity of MIL@MIP could not increase any more in spite of the enhancement of initial concentration of TBM. The well specificity of the MIL@MIP as well as good imprinted effect was demonstrated.

### 3.3. Langmuir Analysis

To evaluate the adsorption characteristic of MIL@MIP, a Langmuir equation for the adsorption balance curve of MIL@MIP was established and shown in Figure 6(b), which regressed into a straight line, and had good linearity (R^2^=0.99). It can be concluded that the adsorption process belonged to the monolayer adsorption on the imprinted surface, and the active site of the adsorbent was located on the surface of the polymer. Once the site was occupied by template molecules, it could not adsorb the other substances again. The saturation adsorption capacity (Q_max_) could be calculated by the slope and intercept of the equation; the values of Q_max_ and KD were 3.571 mg/g and 0.1186 mL/*μ*g, respectively, which was consistent with the experimental data.

### 3.4. Adsorption Kinetics

**Figure 7:**
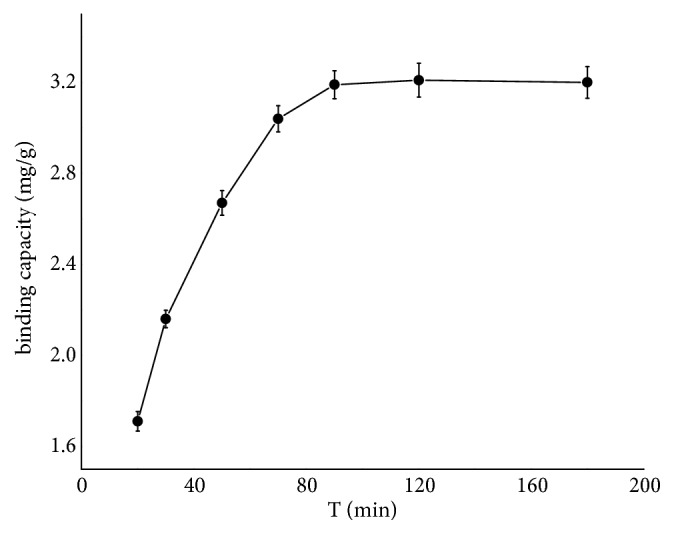
Dynamic adsorptions of MIL@MIP.

As shown in Figure 7, the MIL@MIP maintained high adsorption efficiency for TBM. The adsorption amount increased rapidly and the adsorption amount could reach 53.3% of the Q_max_ in the first 20 minutes. The Q_max_ could be reached within 90 minutes. It was worthwhile to notice that the adsorption saturation time of MIL@MIP would be shortened if the amount of residue was lower. Therefore, the MIL@MIP synthesized in this study would shorten the time of adsorption saturation greatly in the real sample determination. The reason could be that the large surface of MIL-101 can enhance the transfer velocity of the material.

### 3.5. Binding Specificity

**Figure 8:**
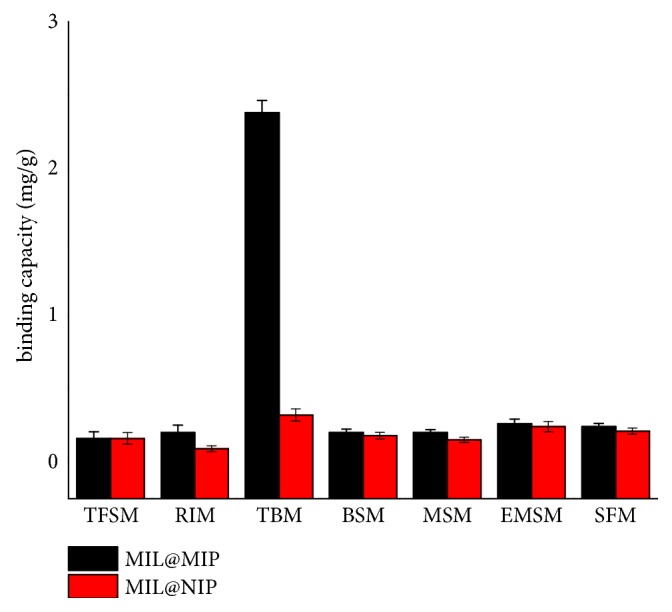
Specific recognition properties of MIL@MIP and MIL@NIP.

The binding specificity test of the MIL@MIP was carried out, followed by six kinds of sulfonylurea pesticides chosen as the function and structure analogues, namely, TFSM, RIM, BSM, MSM, EMSM, and SFM. As shown in Figure 8 and Table 1, it can be concluded that the adsorption capacity of MIL@MIP for TBM is significantly higher than the other six similar substances, and the IF of TBM is also higher than the other six substances, so the MIL@MIP has the strongest affinity for TBM. Comparing the data of Figure 6(a) with those of Figure 8, it can be seen that the IF of TBM in the mixed sample solution is higher than that in the single sample solution because the competitive effect exists among the seven substances in the mixed solution. The adsorption of MIL@MIP decreases less than that of MIL@NIP, because MIL@MIP has imprinted sites that match template molecules very well in shape, size, and spatial distribution.

### 3.6. Optimization of Extraction Conditions

In order to acquire a satisfactory recovery, the influential factors of rinsing solvent and its volume are optimized. The experiment was carried out by enriching 100mL TBM water solution (1.00*μ*g/L), dichloromethane, methyl alcohol, ethyl alcohol, and acetonitrile being chosen as rinsing solvents. The elution effect was expressed by the recovery of TBM. It was discovered that there would be a highest recovery when 8.00mL of acetonitrile was used as rinsing solvent.

### 3.7. Method Validation and Application to Real Samples

**Figure 9:**
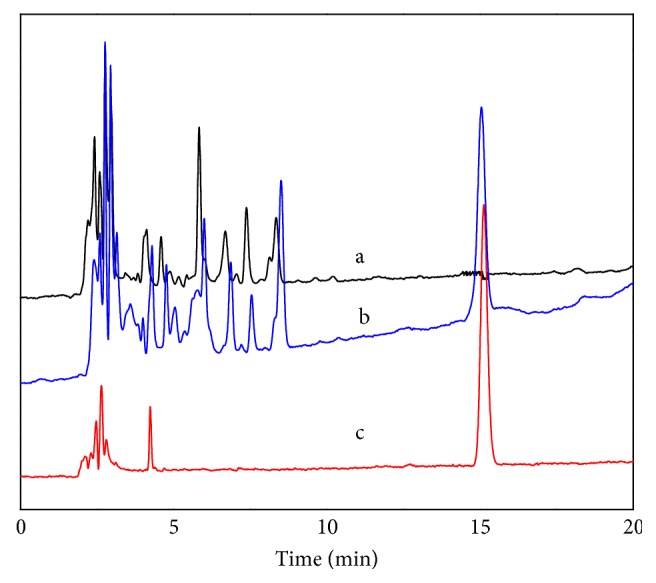
Chromatograms of blank soybean sample (a), soybean sample spiked with TBM (0.045*μ*g/kg) (b), and the standard solution of TBM (c).

The created method was applied to river water, soil, and soybean samples. It was confirmed that the samples were free of TBM. The method accuracy was evaluated by the recovery test with spiked samples. The relative recoveries on MIL@MIP for the spiked water, soil, and soybean were 84.6-92.3%, 93.3-106.7%, and 88.9-93.3%, and the relative standard deviations (RSDs) were 4.8-8.3%, 3.3-6.4%, and 3.5-6.3%, respectively. On the contrary, the relative recoveries on MIL@NIP were very low and they were almost half those of MIL@MIP (Table 2). Limit of detection of the water, soil, and soybean sample was 0.3ng/L, 1.5ng/kg, and 1.5ng/kg (S/N=3), respectively. To further demonstrate the purification effect of the MIL@MIP pretreatment method, 15 anonymous samples, including 5 water samples, 5 soil samples, and 5 soybean samples, were subjected to extract using MIL@MIP sorbents and assayed via the established procedure. Although no positive result was found among the collected samples, the efficiency of the presented MISPE coupled with HPLC method for spiked samples was compared with other reported methods such as HPLC/MS/MS coupled with traditional C_18_ SPE [37] and HPLC method coupled with MIP-SPE [21]. In terms of LOD values and recoveries, the efficiency of this method was better than that of the two other methods. Although a more sensitive MS detector was used in the analysis [37], the high LOD values resulted from the interfering substances because the traditional SPE on C_18_ was not specific and selective to TBM. In the method of HPLC coupled with MIP-SPE [21], although five kinds of SUHs were simultaneously detected using MIP as sorbents, the LOD values were high and the recovery was relatively low, especially to TBM. The low recovery to TBM can be partly attributed to the great difference between its structure and template molecules. However, the low recoveries of the other four substances and the high LOD values may be related to the relative slow mass transfer and small binding capacity. In this paper, the better recoveries and satisfactory LOD values can be achieved by using the MIL-101(Cr)@MIP as sorbent owing to its big binding capacity and selective binding sites on its surface. Furthermore, the method established does not require expensive instrument, consumes much less toxic organic solvent, and has a good clean-up and concentration effect for TBM. All these results revealed that MISPE could effectively remove the interferences and increase the detection ability of TBM (Figure 9) and the HPLC-MISPE method was fast, accurate, and sensitive for detecting the trace TBM in soybean and environmental media.

## 4. Conclusions

In this work, the core–shell molecularly imprinted polymer (MIL@MIP) was synthesized by surface molecular-imprinting technique using MIL-101 as core, which exhibited high adsorption capacity, excellent recognition ability, and high cross-selectivity for TBM. After optimizing the experimental influential factors, the polymer was used as SPE materials and firstly applied to the determination of TBM in river water, soil, and soybeans coupled with HPLC. Moreover, good recoveries and lower LOD values were achieved. It was proved that the developed HPLC-MISPE method was easy and feasible in determination of TBM in samples with complex matrix.

## Figures and Tables

**Table 1:** Competitive loading of TBM and other analogues by MIL@MIP and MIL@NIP.

Analyte	Q_MIP_	Q_NIP_	K_MIP_	K_NIP_	IF	SC
	(mg/g)	(mg/g)	(mL/g)	(mL/g)		
TFSM	0.16	0.16	6.29	6.29	1.00	8.31
RIM	0.20	0.09	9.48	4.24	2.23	3.72
TBM	2.38	0.32	135.07	16.26	8.31	/
BSM	0.20	0.18	10.00	8.99	1.11	7.47
MSM	0.20	0.15	9.09	6.80	1.34	6.22
EMSM	0.26	0.24	15.08	13.90	1.08	7.66
SFM	0.24	0.21	11.08	9.68	1.14	7.26

**Table 2:** Recoveries of TBM on MIL@MIP and on MIL@NIP for spiked water, soil, and soybeans samples.

sample	Spiked Conc. (*μ*g/L, *μ*g/kg×10^−3^)	Determined Conc.		Repeatability		Recovery	
		(*μ*g/L, *μ*g/kg×10^−3^)		(RSD%, n=3)		(%)	
		MIP	NIP	MIP	NIP	MIP	NIP
water	13.0	12.0	5.3	4.8	4.2	92.3	40.8
	6.5	5.9	2.5	6.8	7.2	90.8	35.4
	1.3	1.1	0.7	8.3	6.1	84.6	53.8
soil	45.0	42.0	16.7	3.3	3.9	93.3	37.1
	22.5	24.0	10.2	4.1	5.4	106.7	45.3
	4.5	4.3	2.1	6.4	5.9	95.6	46.7
soybean	45.0	42.0	21.0	3.5	3.8	93.3	46.7
	22.5	20.0	8.7	3.9	4.1	88.9	38.7
	4.5	4.1	2.3	6.3	7.4	91.1	51.1

Water: *μ*g/L; soil and soybean: *μ*g/kg; MIP: MIL@MIP; NIP: MIL@NIP.

## Data Availability

The data used to support the findings of this study are included within the article.
